# Chronic cranial window with access port for repeated cellular manipulations, drug application, and electrophysiology

**DOI:** 10.3389/fncel.2014.00379

**Published:** 2014-11-11

**Authors:** Christopher J. Roome, Bernd Kuhn

**Affiliations:** Optical Neuroimaging Unit, Okinawa Institute of Science and Technology Graduate UniversityOnna-son, Okinawa, Japan

**Keywords:** imaging, *in vivo*, access port, chronic cranial window, two-photon, silicone plug

## Abstract

Chronic cranial windows have been instrumental in advancing optical studies *in vivo*, permitting long-term, high-resolution imaging in various brain regions. However, once a window is attached it is difficult to regain access to the brain under the window for cellular manipulations. Here we describe a simple device that combines long term *in vivo* optical imaging with direct brain access via glass or quartz pipettes and metal, glass, or quartz electrodes for cellular manipulations like dye or drug injections and electrophysiological stimulations or recordings while keeping the craniotomy sterile. Our device comprises a regular cranial window glass coverslip with a drilled access hole later sealed with biocompatible silicone. This chronic cranial window with access port is cheap, easy to manufacture, can be mounted just as the regular chronic cranial window, and is self-sealing after retraction of the pipette or electrode. We demonstrate that multiple injections can be performed through the silicone port by repetitively bolus loading calcium sensitive dye into mouse barrel cortex and recording spontaneous cellular activity over a period of weeks. As an example to the extent of its utility for electrophysiological recording, we describe how simple removal of the silicone seal can permit patch pipette access for whole-cell patch clamp recordings *in vivo*. During these chronic experiments we do not observe any infections under the window or impairment of animal health.

## Introduction

Chronic cranial windows (Levasseur et al., 1975; Grinvald et al., 1991; Holtmaat et al., 2009) have been instrumental in advancing *in vivo* optical imaging studies, permitting long term high resolution imaging in various brain regions. Their success in small animals like mice has been largely due to the simplicity and reliability of the technique (Trachtenberg et al., 2002; Holtmaat et al., 2005, 2009). A single surgical procedure with fast recovery of the experimental animal allows a variety of *in vivo* optical imaging techniques on a single specimen over an extended period of time, potentially longer than 1 year. Meanwhile behavioral training and external stimuli may be implemented and a wealth of data collected from sub-cellular compartments, individual neurons, or neural networks, in anesthetized and awake animals (Holtmaat et al., 2005, 2006; Portera-Cailliau et al., 2005; Mank et al., 2008; Andermann et al., 2010; Adam and Mizrahi, 2011; Kuhn et al., 2012; Xu et al., 2012).

*In vivo* electrical recoding, whole cell recording in particular (Margrie et al., 2002), has likewise resulted in many new insights of how neurons behave in their natural environment. With acute cranial windows it was possible to combine two-photon imaging (Denk et al., 1990; Helmchen and Denk, 2005) and electrophysiology via targeted and shadow patch clamp techniques (Komai et al., 2006; Kitamura et al., 2008). Direct physical access to the brain through acute craniotomies has also been crucial for validating observations made *in vitro* and for calcium sensitive dye imaging or local pharmacological cellular manipulation (Svoboda et al., 1997; Sullivan et al., 2005; Dombeck et al., 2009; Hoogland et al., 2009; Smith et al., 2013). However, these experiments were all terminal.

Techniques that combine chronic imaging and direct cellular recording or manipulation require sophisticated surgeries and window designs (Arieli and Grinvald, 2002) that typically involve dural replacement or covering by a transparent yet electrode penetrable material (Yousef et al., 1999; Shtoyerman et al., 2000; Arieli et al., 2002; Slovin et al., 2002). These windows were mainly applied in larger mammals, but not in mice due to their size.

Despite the utility of these techniques, a simple device that combines chronic imaging with easy physical access to the brain is not currently available. After an initial surgery, such a device would permit multiple cellular manipulations for weeks or months, for drug, dye, DNA, RNA and virus delivery or for sample extractions, and electrophysiological recording and stimulation, while preventing any contamination of the brain and keeping the animal in a healthy condition.

Here, we present a simple method which allows the above manipulations through a silicone plug in the glass window cover slip. We show its utility by repeated injections over a period of several weeks, functional imaging, and electrical recordings by targeted whole-cell patch clamp technique after plug removal.

## Methods and results

A chronic cranial window with access port or silicone plug was developed to meet the following criteria: the device must improve upon similar methods by simplifying the surgical procedure; each window being easy to fabricate and fully adaptable to a typical chronic cranial window surgical procedure; the window adaptation should not degrade the imaging quality, stability or surgical integrity over long periods of repetitive use (weeks to months); it must allow easy, visually guided, and repeatable access to the brain via pipette or electrode for delivery of substance, electrical stimulation or recording.

All animal experiments were approved by the OIST Institutional Animal Care and Use Committee (IACUC) in an Association for Assessment and Accreditation of Laboratory Animal Care (AAALAC International) accredited facility.

### Preparation of the chronic cranial window with access port

**Figure 1:**
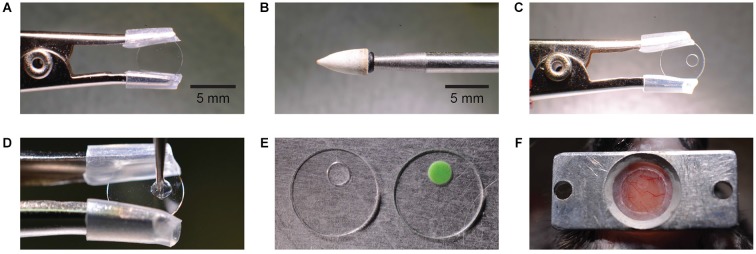
**Manufacturing and mounting of a chronic cranial window with access port**. A round glass cover slip is clamped by a crocodile clamp with silicone tube cover **(A)**. A conical stone drill bit **(B)** is used to grind a circular hole off-center into the glass cover slip **(C)**. The hole is filled with silicone which is applied by a metal drill bit **(D)**. Different silicone types have different features like transparency and stiffness **(E)**. The image shows a Sylgard (left) and a Kwik-Cast (right) filled access port. The window is mounted on the skull taking into account the access direction at the imaging setup **(F)**.

Prior to the surgery the glass window with silicone access port was prepared (Figure 1). A typical circular 5 mm diameter glass coverslip (thickness 170 µm) was clamped using a crocodile clip with silicone tubing covering the teeth to prevent chipping the glass (Figure 1A). A conical shaped stone drill bit (CA1063, Minimo Precision Instruments and Tools, Figure 1B) and a high speed dental drill (OS-40, Osada) was used to drill a hole of approximately 1.5 mm in diameter through the glass with the tip of the bit (Figure 1C). This step took 1–2 min. For a smooth finish around the hole edge and to avoid chipping the glass, the finish drilling was made from both sides of the glass.

After removing any glass dust with compressed air, the drilled hole was sealed with silicone. Three varieties of silicone were tested: Sylgard 184 (Dow Corning Corporation), Kwik-Sil, and Kwik-Cast (W.P.I.INC.), each with its advantages and limitations. Since Sylgard 184 and Kwik-Sil are transparent these were preferred for controling entry of the micropipette through the dura and to avoid piercing blood vessels. Kwik-Cast is partially opaque and so limits visibility and increases background fluorescence. Fabrication of the access port was easier using Kwik-Sil or Kwik-Cast which cure faster (10 min), while Sylgard 184 required additional heating to speed curing and raise its viscosity. We observed that the softer silicone, Kwik-Sil, exerted excessive pressure on the dura during micropipette penetrations, but the more rigid silicone, Sylgard 184 and Kwik-Cast, did not. The additional steps required for curing Sylgard 184 were seen as minor, and so for its transparency and higher rigidity, Sylgard 184 was chosen in our experiments.

In all three cases the silicone is composed of two parts, the elastomer and the curing agent, which must be mixed and allowed to partially cure to raise silicone viscosity and prevent its spreading across the glass surface. To raise Sylgard viscosity the elastomer and curing agent were mixed at a ratio of 10:1 on a glass slide and then heated (while mixing) over a glass bead sterilizer, for approximately 30 s at 80°C. A droplet of silicone is applied to the window hole edges (Figure 1D) using a sterile metal drill bit carefully inserted into the hole. A metal drill bit is used because it sticks less to the silicone than for example, a wooden toothpick.

After retracting the drill bit, a droplet of silicone remains within the window hole and seals it completely. The size of the silicone droplet should be adjusted to create a silicone plug of central thickness 130 ± 20 µm (*n* = 3), so that the surface of the silicone plug lies roughly flat with both sides of the window glass. To keep the silicone clear and evenly distributed within the hole and to prevent it spreading onto the surrounding glass surface it is important that no physical contact is made with the silicone during the curing process. Sylgard 184 requires heating to speed curing. Heating the glass window with silicone again over a glass bead sterilizer for 10 min at 80°C, for example, is sufficient. Kwik-cast and Kwik-sil are fast curing at room temperature and set after approximately 15 min. Once set, the silicone forms a durable, tight, and biocompatible access port through the window, which can then be sterilized with alcohol and stored or immediately used in surgery (Figure 1E).

### Surgery

Chronic cranial window surgeries were performed on female and male C57/BL6 mice, 28- to 56-days old using the 5 mm glass cover slip with silicone access port following the procedure described previously (Holtmaat et al., 2012). Mice were deeply anesthetized throughout the surgery by 1–2% isoflurane and a stereotaxic frame was used to immobilize the head. Eye ointment was applied to the eyes for protection and to prevent dehydration. Carprofen (5 µg/g body weight, intraperitoneal), and Buprenorphine (0.1 µg/g body weight, subcutaneous) were administered to reduce postoperative inflammation and pain, respectively. Dexamethasone (2 µg/g body weight) was also administered by intramuscular injection into the quadriceps muscle to reduce immune response and inflammation. After hair removal by trimmer and hair removal cream, the skin was opened with a scalpel. The skull was cleaned with a lidocaine solution and carefully dried with compressed air. Using a dental drill the area around the intended craniotomy (center at 1.5 mm posterior of bregma, 3.0 mm lateral, diameter 3–4 mm) was thinned.

One notable alteration to the before described procedure was made for skull removal to expose the dura mater. Rather than using forceps to perforate and lift the skull, which can puncture the dura upon insertion (step 9 in Holtmaat et al., 2012), a wooden toothpick was attached vertically to the center of the bone above the intended craniotomy region using dental acrylic. The dental acrylic was allowed to spread over the bone that will be removed and also partly over the thinned bone around the circumference of the craniotomy region (in total about 3.5–4.5 mm in diameter). Once the dental acrylic had set (approx. 15–20 min) the toothpick was gently lifted to separate the skull from the dura, which would crack evenly along the edge of the cemented area. We found this technique reliable in creating a clean bone edge around the craniotomy and in preventing dural bleeding, both essential for creating a clear and closely fitting cranial window. This technique increased our surgery success rate to 80% (*n* = 40).

It was not necessary to apply agarose between glass and dura or to remove the dura; the glass window was mounted directly onto the dura and sealed with super glue to the skull as in a typical chronic cranial window surgery. After allowing the superglue to set (1–2 min), a rectangular aluminum headplate (Figure 1F) was positioned over the glass window and attached by applying dental acrylic using a toothpick. The dental acrylic was allowed to fill all spaces under and around the headplate including the edges of the glass window and also cover all areas of exposed skull. When positioning the glass window, it is important to position the access port appropriately for its intended use. The access port must be positioned over the location where injections are to be made and adjacent to the location where imaging is to be performed. Special care must be taken to ensure that the intended injection and imaging areas are clear of blood vessels, which are visible through the silicone plug (Figure 1F). The surgery takes about 1 h. If more time was needed saline (10–20 µl/g body weight) was injected subcutaneously to avoid dehydration. The mouse was allowed to recover from surgery at least 2 days.

### Imaging setup

We used a custom-built combined wide-field and two-photon microscope (MOM, Sutter, designed by Winfried Denk) with either a 5×/N.A. 0.13 air objective (Zeiss) or a 25×/N.A. 1.05 water immersion objective with 2 mm working distance (Olympus). The collar of the 25× objective was adjusted to correct for the window glass thickness (170 µm). Brightfield imaging was done with a sCMOS camera (PCO.edge, PCO) in the green spectral range (475 nm–575 nm, Chroma). A femtosecond-pulsed Ti:sapphire laser was used to excite fluorescence which was detected by two GaAsP photomultiplier tubes (Hamamatsu) in the spectral range of 490 nm–595 nm (green) and 570 nm–640 nm (red) (Chroma).

### Injections through access port

**Figure 2:**
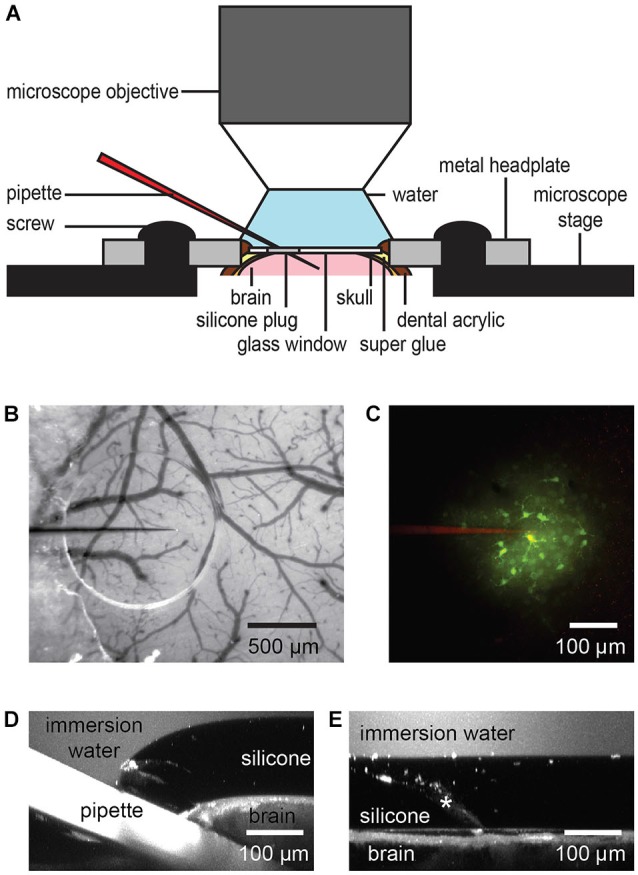
**Dye or drug application through the port of a chronic cranial window**. The silicone plug can be easily penetrated by a glass or quartz pipette taking into account the steric limitations of the imaging setup like working distance and size of the objective lens **(A)**. A transparent silicone plug allows avoiding blood vessels **(B)**. The image was taken before entering the brain. Two-photon imaging shows bolus loading after penetration of the silicone plug **(C)**. A *z*-axis projection through the injection site shows the pipette position and silicone/dura displacement during a typical pipette injection **(D)**. Here,1 µM fluorescein was added to the water immersion for visualization. Following pipette retraction the silicone and dura return to their original positions (**E**, injection track indicated by asterisk), even after multiple injections.

The window access port permitted repetitive and targeted access to the brain for application of drugs or other compounds, like dyes or viral vectors (Figure 2) and re-sealed when the pipette was retracted.

A glass or quartz pipette was beveled with a course diamond abrasive plate (BV-10, Sutter) to a defined opening between 5 and 15 µm. Beveling creates a sharp edge at the tip of the pipette and reduces clogging. After beveling, the pipette was filled with the solution which was delivered, mounted on a pipette holder on a micromanipulator, and connected to tubing that allows pressure application to the inside of the pipette.

After head-fixing the mouse on the microscope stage the beveled pipette was controlled and advanced diagonally by micromanipulator through the silicone plug under visual control using bright field (Figure 2B) or fluorescence microscopy (Figure 2C). An angle of 22–27° to horizontal of the pipette holder allowed the usage of large 2 mm working distance objectives, such as the Olympus 25×/N.A. 1.05 objective. The location of the injection site and angle of the pipette should be adjusted in accordance with the intended depth of injection and to prevent imaging from being obscured by the silicone/glass interface, the pipette tip (target imaging region) should be located typically more than 500 µm from this interface.

Blood vessels should be avoided during the injections. With a transparent silicone plug it is possible to visualize blood vessels in the port area and to follow the micropipette position during entry through the silicone, the dura and into the brain. An accidental puncture of a blood vessel can terminate the experiment. This occurred on two occasions during our long term experiments. A total of 25 injections were made, giving an injection success rate of roughly 90%. In general, either a green filter in front of the camera or green light for illumination increases blood vessel to neuronal tissue contrast.

After reaching the target area, 14 mbar of pressure was applied to the pipette for 1 min to deliver the dye (Figure 2C). Typical injection pressure was in the range of 0.7–70 mbar (0.01–1.0 PSI). This can be done, dependent on the application, for milliseconds to hours. Finally, the micropipette was retracted and the silicone plug re-sealed to maintain sterile conditions in the brain. During our injections the maximum vertical displacement of the dura under the inserted pipette was found to be 107 ± 17 µm (*n* = 6; Figure 2D). Upon micropipette retraction the silicone returned immediately to its original position and did not permanently deform or separate from the glass, even after multiple injections (Figure 2E).

### Chronic functional imaging after repeated dye injections

To verify the reliability and repeatability of the technique, we repetitively bolus-loaded (Stosiek et al., 2003) cortical layer 2/3 of barrel cortex with a calcium sensitive dye, Oregon Green 488 BAPTA-1 AM (OGB-1 AM, Invitrogen) and recorded spontaneous cellular activity from lightly anesthetized mice (0.5% Isoflurane). For bolus loading we used beveled quartz pipettes (1 mm outside diameter, 0.7 mm inside diameter and 5 µm tip diameter), backfilled with 100 µM OGB-1 AM, 0.02% Pluronic F-127 (Invitrogen), 0.1% DMSO (Invitrogen) dissolved in a pipette solution containing, in mM: 150 NaCl, 2.5 KCl, 10 HEPES, pH 7.4. For pipette visualization, Alexa 594 (10 µM, Invitrogen) was also included in the pipette solution (Figure 2C).

**Figure 3:**
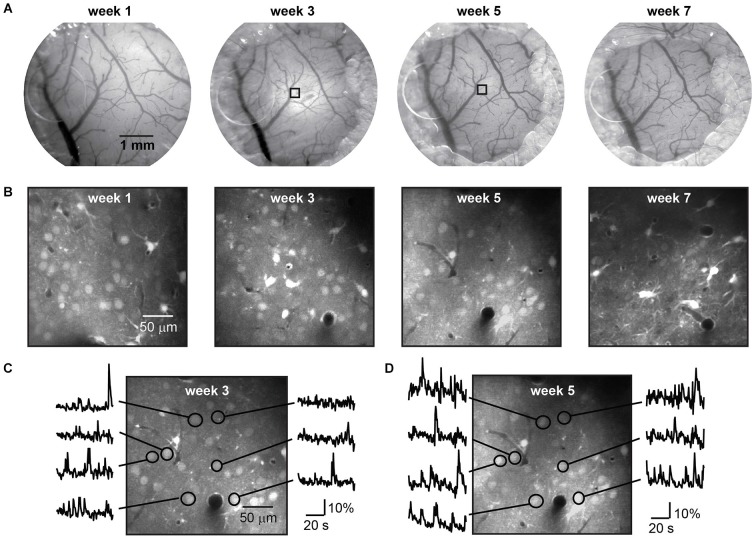
**Long term imaging with repeated dye loading**. Repeated bright field imaging of blood vessels during a 7 week period **(A)** starting 1 week after mounting of the chronic cranial window with access port. Regrowth of bone and gradual hardening of the dura but not infection terminated the time course. The decreasing diameter of the craniotomy due to regrowth of bone can be seen from left to right. During this period eight injections of the calcium dye OGB-1 AM-ester were performed, four of which are shown in **(B)**. Functional signals imaged with two-photon microscopy at a depth of 250 µm below the dura in a lightly anesthetized mouse (0.5% isoflurane) are shown in two recordings after repeated injection of AM-ester dye to the same location, indicated in **(A)** by squares, three **(C)** and five **(D)** weeks after mounting the window.

The dye was loaded up to eight times over a period of 7 weeks (five mice), without impairment of the window clarity (Figure 3A) or imaging quality (Figure 3B). Following each injection a period of 1 h was given for dye uptake into the cells and spontaneous activity was recorded in the same imaging plane during several sessions (Figures 3C,D). Imaging data was registered to compensate for movement artifacts, then regions of interest were selected, time traces background-corrected, and Δ*F/F* calculated.

Imaging regions were selected based upon optimal cellular labeling and imaging; those with the brightest labeled cells, unobscured by blood vessels at the surface. On one occasion we imaged from identical neurons labeled with OGB-1 on different weeks demonstrating that no adverse effects on cellular function or image quality had occurred locally, following a previous injection (Figures 3C,D).

During the experimental period all mice showed normal behavior and gave no signs of distress. As with regular chronic cranial windows, regrowth of bone became evident around the window perimeter. The extent of bone regrowth varied between mice. In some mice no bone regrowth was observed over multiple weeks. In mice where bone regrowth was apparent the regions of fastest bone regrowth were randomly distributed and independent of access port position. The maximum rate of bone regrowth in these regions was 250 ± 109 µm/week (*n* = 15 samples from five mice), and was independent of the number of weeks following the craniotomy. Under the plug we found a gradual hardening of the dura which eventually impaired imaging quality and prevented simple access to the brain after week 7 (Figures 3A,B right). The regrowth of bone can be overcome or at least delayed by an increased diameter of the craniotomy.

### Electrophysiology

**Figure 4:**
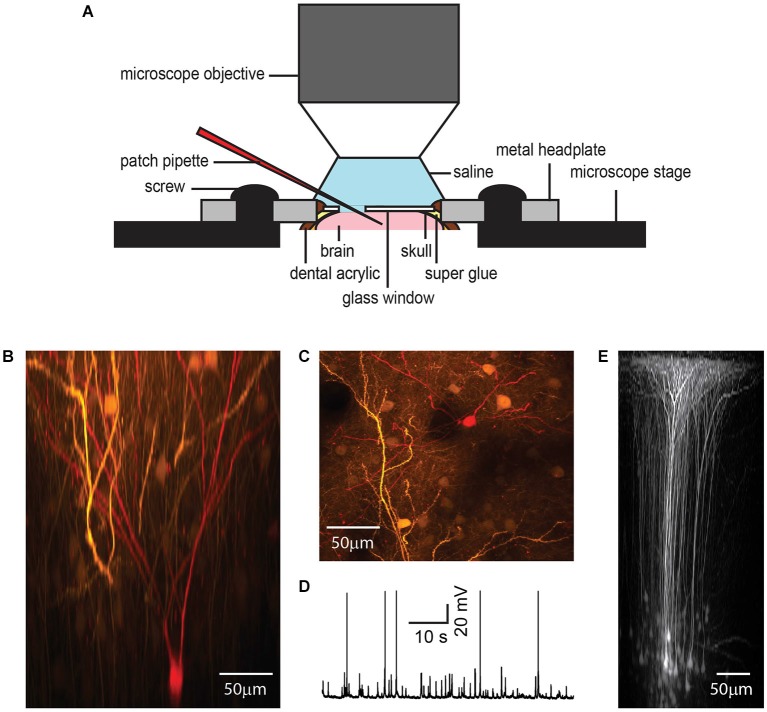
**Chronic cranial window with access port for electrophysiology**. While metal electrodes and beveled electrodes for single or multi-unit recording can simply penetrate the silicone plug, in our hands, it was necessary to remove the plug for patch clamp experiments to reach a high success rate **(A)**. A GFP-expressing layer 2/3 pyramidal neuron shown in *x-z* plane projection **(B)** and *x-y* plane projection **(C)** was targeted for whole-cell patch clamp recording under anesthesia (1% isoflurane) **(D)** and filled with a red fluorescent dye after plug removal. The access port does not affect the imaging quality as shown by *in vivo* imaging of GFP-expressing neurons in layer 5 of barrel cortex after viral gene transfer **(E)**.

As a second demonstration of how the chronic cranial window with access port may be applied, we conducted targeted whole cell patch clamp recordings (*n* = 3) on layer 2/3 pyramidal cells previously labeled with GFP (Figure 4).

An Adeno-Associated Viral vector (AAV2/1-hSYN-GFP, UPenn Vector Core Facility) was injected to transfect layer 2/3 (Figures 4B,C) or layer 5 pyramidal cells (Figure 4E) to express GFP. In this case the injection procedure was identical to the bolus loading technique with the exception that the AAV was injected over a longer period (30 min–1 h) using lower pipette pressure (<0.7 mbar, 10 µm pipette opening, about 10 nl injection volume) in order to focus the GFP transfection to the immediate injection region and reduce excessive labeling. Green fluorescent protein expression was monitored over several days before beginning the electrophysiological recordings.

After suitable expression of GFP in neurons, we performed targeted-patch whole-cell recordings from the GFP labeled neurons in layer 2/3 in anesthetized mice (1% isoflurane). For successful patch clamp recordings it is essential to maintain a clear pipette tip using high positive pressure to continually blow debris away from the tip with the internal solution. While it was possible to penetrate the silicone and dura with regular glass patch pipettes for loose patch recordings, we were not able to reach the GOhm seal necessary for whole cell patch clamp recording. This is probably because the continual flow of internal solution that maintains a clear pipette tip, is halted during silicone penetration. Thus, in our hands, it was necessary to remove the silicon plug covering the access hole for reliable whole cell patch clamp recording. The silicone plug was removed using a 26G hypodermic needle and forceps under a microscope while the mouse is anesthetized (1% isoflurane) and headfixed in a stereotax. Removal of the silicone was relatively easy and quick to do, although care is necessary to avoid puncturing the dura. A droplet of sterile saline (0.9% NaCl) was then added to the window glass covering the exposed dura.

The anesthetized mouse was transferred to the microscope stage and a ground electrode positioned in the saline covering the glass and dura. Once a GFP labeled layer 2/3 neuron had been identified as a target, a patch electrode with tip resistance 5–8 MOhm containing internal solution (in mM): 100 K-gluconate, 20 KCl, 10 HEPES, 4 MgATP, 0.3 Na_2_GTP, 10 Na-Phosphocreatine, 0.05 Alexa 594, pH 7.3, 290 mOsm), was advanced diagonally through the dura, while taking care to avoid blood vessels, for whole-cell targeted patch clamp recording (Komai et al., 2006). Cellular activity in the anesthetized mouse was recorded in current clamp mode (EPC10 USB, HEKA) (Figure 4D). Recordings remained stable for up to 40 min, during which time the patched neuron was filled with Alexa Fluor 594 (50 µM; Invitrogen) for later 3D reconstruction.

In general, dura penetration with a patch pipette after plug removal is not as smooth as in an acute experiment. This might be due to some thickening of the dura in response to the skull removal. However, for whole-cell patch clamp electrophysiology we found it was not necessary to remove the dura, although a wide shafted patch pipette may be used initially to create a hole in the dura to aid penetration of subsequent patch pipettes. Alternatively the dura can be slightly opened by making a small cut for easier access.

Following the electrophysiological recording the window access port was resealed by gently drying the dura with absorbent swabs (Sugi Saugtupfer, Kettenbach GmbH and Co. KG) and compressed air. The hole opening was then re-filled with fast curing (10 min) silicone sealant such as Kwik-Sil or Kwik-Cast using the same method as described for the access port fabrication. For Kwik-Sil the plug was again transparent and would allow for imaging. However, after repeated plug removal and penetration of the dura with rather wide patch pipettes, increased scar formation or inflammation has to be expected.

We also successfully performed electrical stimulation of dendrites with 0.1 and 1.0 MOhm tungsten electrodes and recorded optically dendritic responses. Penetration of the plug and dura was easy using tungsten electrodes.

Sharp glass microelectrodes however were prone to bending and breaking and so stiffer quartz glass microelectrodes (preferably beveled) are a better alternative for electrical sharp recording and/or stimulation.

## Discussion

We have described and demonstrated a device that permits long term *in vivo* optical imaging and combines access to the brain for cellular manipulations. A biocompatible silicone plug was incorporated into a typical chronic cranial window to allow access to the brain via micropipettes or electrodes. After each injection the silicone plug reseals, in a similar way to the silicone container caps used in medicine, where surgical syringes repetitively penetrate the cap but the cap maintains its seal and the liquid inside the container remains sterile.

The device was designed to satisfy these criteria: the device should be easy to fabricate and fully adaptable to a typical chronic cranial window surgical procedure: the device should not degrade the imaging quality, stability, or surgical integrity, for long periods of repetitive use (weeks to months) and: the device should allow easy, visually guided, and repeatable access to the brain via pipette or electrode, for delivery of substance, electrical stimulation or recording at a single cell resolution.

This window with access port can be fabricated from a typical glass coverslip and requires no sophisticated equipment or materials. Each window can be prepared immediately prior to surgery taking no more than 30 min, and the surgical procedure is identical to a typical chronic cranial window surgery. Experiments were repeated eight times per mouse during a 7 week period during which time we saw no abnormal behavior or signs of distress in the mice, and functional *in vivo* recordings showed very little change in quality over successive weeks.

In general, the shape and size of the glass window as well as that of the access hole (or several holes) can be adapted to meet the specific needs of the experiment. For example, two off-center access ports can be used to locally perfuse brain tissue with one pipette as a source and one as a drain while imaging the perfused tissue (as in Engert and Bonhoeffer, 1999). Multiple access ports can be used, for example to electrically stimulate and record while applying a drug *in vivo*.

For patch experiments, we needed to remove the plug to achieve a reliable GOhm seal. However, electrical recordings where no GOhm seal is needed can be performed without plug removal. This includes single and multi-unit recordings with slightly beveled sharp electrodes or metal electrodes like tungsten electrodes. Fine glass electrodes tend to bend and slide on the silicone surface under shallow angle approach. This problem can be overcome by beveling and by using quartz pipettes or pipettes with wider shanks.

We believe this device would be valuable across a broad range of *in vivo* studies in various species, the main targets being neurons, glia, and brain vasculature for *in vivo* imaging and cellular manipulation. Because of its biocompatibility and stability, the device guarantees the sterile conditions and the lowest possible immune response that are essential for optimal optical imaging.

Using this technique animals recover quickly from surgery and can be used repetitively for weeks or months, such that the number of animals used in research can be significantly reduced, while the information gained from a single animal can be dramatically increased. This low-cost simple device will simplify current experiments and, even more importantly, allow many new experiments that because of limited brain access have not yet been possible. This is particularly true for *in vivo* drug screening, for example, where a time course of local drug application to the brain over several months can now be done in a single animal while the effects of the drug can be repeatedly monitored during this time. Using current techniques, many animals would be sacrificed at different time points for the same study. Possibly the most significant advantage of this device is that *in vivo* cellular manipulation and/or electrophysiological recording can now be easily performed, repeatedly, and in combination with long term optical imaging; an ever growing necessity for *in vivo* studies in neuroscience.

## Conflict of interest statement

US patent application Kuhn, B., and Roome, C. J. (2013) Chronic cranial window allowing drug application and electrophysiology US61/918,193.
